# Selective Utility of Periodic Acid-Schiff Staining in Sinonasal Masses: A Cross-Sectional Observational Study

**DOI:** 10.7759/cureus.113971

**Published:** 2026-08-04

**Authors:** Erwin M Toppo, Sunil K Mahto, Saurav Banerjee, Anshu Jamaiyar, Manoj K Paswan, Arvind Kumar

**Affiliations:** 1 Pathology, Rajendra Institute of Medical Sciences, Ranchi, IND

**Keywords:** ancillary stain, fungal rhinosinusitis, histopathology, mucin, nasal polyp, periodic acid-schiff stain, sinonasal mass

## Abstract

Introduction

Sinonasal masses encompass inflammatory, infective, benign neoplastic, and malignant lesions with overlapping clinical and microscopic features. Periodic acid-Schiff (PAS) staining can demonstrate carbohydrate-rich mucin, basement membrane material, and fungal cell walls, but its incremental value when applied routinely to all sinonasal specimens is uncertain. This study evaluated the component-specific and confirmatory utility of PAS staining in routine sinonasal histopathology.

Methods

This prospective, single-center, cross-sectional observational study included 162 consecutive eligible sinonasal biopsy and resection specimens received from March 2025 to December 2025. Routine hematoxylin and eosin (H&E) sections were examined before standard PAS staining. Demographic data, site, broad histological category, necrosis, morphologically localized PAS-reactive mucin, fungal elements, and the effect of PAS on interpretation were recorded. Fisher's exact test was used to assess the association between necrosis on H&E and fungal demonstration by PAS.

Results

Patients ranged from eight to 75 years of age, with a mean age of 31.5 years (standard deviation: 18.1). The nasal cavity was involved in 151 cases (93.2%). Non-neoplastic lesions accounted for 128 cases (79.0%), benign neoplasms for 22 (13.6%), and malignant neoplasms for 12 (7.4%); inflammatory nasal polyp was the most frequent individual diagnosis (110 cases). PAS-reactive mucin was identified in 119 cases (73.5%) but did not independently modify the final histopathological interpretation. Fungal elements were demonstrated by PAS in all 19 cases with prior H&E-based suspicion and in none of the 143 cases without preceding H&E suspicion. These findings reflect confirmatory agreement within the routine sequential diagnostic workflow and should be interpreted as evidence of the supportive role of PAS rather than its independent diagnostic performance. Among 31 cases with necrosis, 19 (61.3%) showed fungal elements, compared with none of the 131 cases without necrosis (Fisher's exact p<0.001). PAS did not substantially alter the broad histological category but provided confirmatory support in cases with morphologically suspected fungal infection.

Conclusion

Routine PAS staining of every sinonasal specimen had limited additional diagnostic contribution in this cohort. As PAS evaluation was performed after H&E review, its independent screening performance could not be assessed. Its main utility was targeted confirmation and enhanced visualization of fungal organisms in morphologically suspicious cases, particularly those with necrosis. PAS should therefore be used as a selective ancillary stain guided by routine histological findings rather than as a universal screening stain for all sinonasal specimens.

## Introduction

Sinonasal masses are a heterogeneous group of inflammatory, infective, benign neoplastic, and malignant lesions arising in the nasal cavity and paranasal sinuses. Patients commonly present with nasal obstruction, discharge, epistaxis, hyposmia, anosmia, or a visible polypoidal growth. Although these presentations overlap, management differs substantially across chronic inflammatory disease, fungal infection, benign neoplasia, and malignancy, making histopathological classification essential [1-4].

Endoscopic biopsies are often small, fragmented, ulcerated, inflamed, or necrotic. These changes can obscure sparse fungal elements, mucin, basement membrane material, and glandular architecture. Inflammatory polyps may also show goblet-cell hyperplasia, prominent glands, edema, and secondary epithelial alterations that create diagnostic overlap with selected glandular or papillary lesions [3-6].

The periodic acid-Schiff (PAS) reaction oxidizes vicinal glycol groups to aldehydes, which react with Schiff reagent to produce a magenta reaction product. In tissue sections, PAS highlights neutral carbohydrate-rich mucosubstances, glycogen, basement membranes, and fungal cell walls [7]. In fungal rhinosinusitis, special stains may improve visualization of organisms within necrotic debris or allergic mucin, although detection can be influenced by the quantity and processing of mucin [8-11]. Allergic fungal rhinosinusitis is a non-invasive form characterized by eosinophil-rich allergic mucin containing fungal elements, whereas invasive forms are defined by tissue or vascular invasion [8,12-14]. Histomorphological patterns may provide additional clues to invasive fungal disease, but organism morphology, host response, and clinical and microbiological correlation remain important for final interpretation [9,10,15].

Despite the technical simplicity and wide availability of PAS, routine application to every sinonasal biopsy may provide little additional information when H&E morphology is already diagnostic. The present study therefore evaluated the component-specific findings and incremental or confirmatory contribution of PAS staining across consecutive sinonasal mass specimens, with particular emphasis on fungal demonstration and mucin visualization.

## Materials and methods

Study design and setting

This prospective, single-center, cross-sectional observational study was conducted in the Department of Pathology, Rajendra Institute of Medical Sciences (RIMS), Ranchi, India. Consecutive sinonasal specimens received for routine histopathological examination between March 2025 and December 2025 were included.

Study population

Patients of any age and either sex undergoing biopsy or surgical resection for clinically suspected sinonasal masses were included. Cases with inadequate clinical information, insufficient or poorly preserved tissue, duplicate specimens, or treatment-related artifacts precluding histopathological evaluation were excluded. A total of 162 specimens were analyzed.

Sampling technique and sample size calculation

A consecutive non-probability sampling technique with total enumeration was used. All eligible sinonasal biopsy and resection specimens received during the predefined study period were included. The minimum sample size was estimated using the following single-proportion formula.

$n = \frac{Z^2 P(1 - P)}{d^2}$

Here, n is the required sample size, Z is the standard normal deviate corresponding to the desired confidence level, P is the anticipated prevalence, and d is the desired absolute precision. A 95% confidence level was used (Z=1.96). During study planning, published estimates regarding PAS positivity, fungal detection, or the incremental diagnostic yield of PAS staining in unselected sinonasal specimens were not available. Therefore, the anticipated prevalence was taken as 4% (p=0.04), based on the population-based study by Hedman et al., which reported a 4.3% prevalence of nasal polyposis [16]. The desired absolute precision was 3% (d=0.03). Substitution of these values yielded a calculated minimum sample size of 163.9, which was rounded to 164 cases.

During the predefined study period, 162 eligible specimens were available, and all were included in the final analysis, representing 98.8% of the calculated target sample size. The slight shortfall from the calculated target sample size reflected the finite number of eligible cases available during the predefined study period and is unlikely to have materially affected the overall study findings. The sample size was determined during study planning using the best available epidemiological data. Although it was adequate for the overall study cohort, it was not specifically calculated to provide statistical power for subgroup analyses of fungal lesions. Accordingly, findings from the fungal subgroup should be interpreted with appropriate caution.

Histopathological evaluation and PAS staining

All specimens were routinely processed and stained with hematoxylin and eosin (H&E). Lesions were categorized as non-neoplastic, benign neoplastic, or malignant neoplastic. PAS staining was performed on paraffin sections using the routine laboratory protocol, with kidney tissue as the positive control. H&E and PAS slides were reviewed jointly by the postgraduate investigator and supervising pathologist, with disagreements resolved by consensus. PAS slides were reviewed with knowledge of the corresponding H&E findings as part of the routine diagnostic workflow; therefore, PAS assessment was not performed in a blinded manner. Clinical history was not available to the reviewers during PAS assessment. PAS findings were evaluated for their additional confirmatory contribution to mucin characterization, basement membrane and glandular pattern evaluation, and detection of fungal elements. Cases were categorized based on the H&E impression and PAS findings into the following four groups: (1) fungal elements suspected on H&E and confirmed by PAS, (2) fungal elements suspected on H&E but not demonstrated by PAS, (3) no fungal suspicion on H&E but fungal elements identified on PAS, and (4) no fungal suspicion on H&E with negative PAS findings. The contribution of PAS was assessed as confirmatory when it supported the H&E impression and as additional diagnostic information when it provided findings not evident on H&E.

Statistical analysis

Data were analyzed using Jamovi (New South Wales, Australia: Sydney) and Microsoft Excel (Redmond, WA: Microsoft Corp.). Continuous variables are presented as mean±standard deviation and categorical variables as frequencies and percentages. Fisher's exact test was used to assess the association between necrosis on H&E and fungal detection by PAS. A two-sided p<0.05 was considered statistically significant. Diagnostic accuracy measures were not calculated because an independent reference standard was unavailable.

## Results

Clinicopathological characteristics

A total of 162 sinonasal mass specimens were included. Patient age ranged from eight to 75 years, with a mean of 31.5 years (SD: 18.1). The 20-39 year age group was the largest subgroup, comprising 59 cases (36.4%). There were 95 male patients (58.6%) and 67 female patients (41.4%). The nasal cavity was the recorded site in 151 cases (93.2%), multiple sinonasal sites were involved in 10 cases (6.2%), and one case (0.6%) had an isolated maxillary sinus site.

Non-neoplastic lesions formed the largest histological category, accounting for 128 cases (79.0%). Benign neoplasms accounted for 22 cases (13.6%) and malignant neoplasms for 12 cases (7.4%). Inflammatory nasal polyp was the most frequent individual diagnosis, with 110 cases. The study was designed to evaluate the confirmatory contribution of PAS rather than the epidemiological distribution of individual sinonasal lesions; therefore, lesion-wise subgroup frequencies were not analyzed further. The baseline clinicopathological characteristics of the study cohort are summarized in Table 1.

**Table 1:** Baseline clinicopathological characteristics of the study cohort (n=162). Age range: eight to 75 years; mean age: 31.5 years; standard deviation: 18.1 years.

Characteristic	Category	Number of cases	Percentage
Age group (years)	<20	54	33.3
	20-39	59	36.4
	40-59	38	23.5
	≥60	11	6.8
Sex	Male	95	58.6
	Female	67	41.4
Site	Nasal cavity	151	93.2
	Multiple sinonasal sites	10	6.2
	Maxillary sinus	1	0.6
Histological category	Non-neoplastic	128	79
	Benign neoplastic	22	13.6
	Malignant neoplastic	12	7.4

PAS findings

Morphologically localized PAS-reactive mucin was identified in 119 of 162 cases (73.5%). PAS accentuated surface goblet-cell mucin, glandular luminal secretions, and selected basement membrane material. These findings improved visualization but did not provide additional diagnostic information sufficient to modify the H&E-based interpretation in non-fungal cases.

Fungal elements were demonstrated by PAS in 19 cases (11.7%). All 19 cases had already raised morphological suspicion for fungal infection on H&E, whereas none of the 143 cases without H&E suspicion showed fungal organisms on PAS. This complete concordance is reported descriptively and reflects confirmatory agreement within the routine unblinded diagnostic workflow. Accordingly, these findings should be interpreted as demonstrating concordance between H&E-based morphological suspicion and PAS confirmation rather than the independent fungal-detection capability of PAS. It should not be interpreted as evidence of the independent diagnostic performance, sensitivity, or specificity of PAS because PAS assessment was performed with knowledge of the corresponding H&E findings and no independent reference standard was available.

Necrosis was present on H&E in 31 cases. Fungal elements were demonstrated in 19 of these 31 cases (61.3%) and in none of the 131 cases without necrosis. The association between necrosis and fungal demonstration by PAS was statistically significant (Fisher's exact p<0.001). The association between necrosis on H&E and demonstration of fungal elements by PAS is presented in Table 2. The concordance between H&E-based suspicion of fungal disease and PAS demonstration of fungal elements is shown in Table 3.

**Table 2:** Necrosis on H&E and demonstration of fungal elements by PAS. Association between necrosis on H&E sections and demonstration of fungal elements by PAS staining. Fisher's exact test demonstrated a statistically significant association between the presence of necrosis and PAS detection of fungal elements (two-sided p<0.001). PAS: periodic acid-Schiff

Necrosis on H&E	Fungus absent on PAS, n (%)	Fungus present on PAS, n (%)	Total, n
Absent	131 (100)	0 (0)	131
Present	12 (38.7)	19 (61.3)	31
Total, n	143	19	162

**Table 3:** H&E fungal suspicion and demonstration of fungal elements by PAS. PAS: periodic acid-Schiff

Fungal suspicion on H&E	Fungus absent on PAS	Fungus present on PAS	Total
No fungal suspicion, n	143	0	143
Fungal infection suspected, n	0	19	19
Total, n	143	19	162

Confirmatory contribution of PAS

PAS did not substantially alter the broad H&E-based histological category in any case. In the 19 cases with suspected fungal infection, PAS provided additional morphological confirmation of fungal structures and supported the final diagnosis. In the remaining morphological contexts, PAS demonstrated expected carbohydrate-rich material or basement membrane structures but did not provide decisive additional diagnostic information. Cases were grouped according to the principal diagnostic question identified during H&E review and the subsequent contribution of PAS findings. The contribution of PAS according to the principal H&E-based assessment context is summarized in Table 4.

**Table 4:** Contribution of PAS according to the principal H&E-based assessment context. PAS: periodic acid-Schiff

Principal assessment context	Cases, n	Principal PAS observation	Effect on interpretation
Basement membrane or glandular pattern assessment	13	Enhanced visualization of PAS-reactive basement membrane/glandular material	No change in diagnosis
Mucin visualization in a polyp	110	PAS-reactive mucin visualized in morphologically appropriate sites	No change in diagnosis
Evaluation of an ambiguous biopsy	20	No decisive additional component identified	No change in diagnosis
Suspected fungal infection	19	Fungal structures confirmed in all 19 cases	Confirmatory support for final diagnosis

## Discussion

The principal finding was that routine PAS provided limited additional diagnostic contribution across an unselected series of sinonasal masses. Most cases were adequately classified on H&E, and PAS did not change the broad histological category. Its clearest contribution was confirmation of fungal structures in 19 cases suspected on routine morphology. These findings support selective rather than routine PAS use.

Non-neoplastic lesions predominated, and inflammatory nasal polyp was the most frequent diagnosis, consistent with Indian series in which inflammatory lesions outnumber neoplasms [1,2]. Male predominance was observed, but occupational, environmental, behavioral, and healthcare-access factors were not assessed. Therefore, its cause cannot be inferred.

Fungal elements were demonstrated in 19 of 31 specimens with necrosis and in none of 131 specimens without necrosis. Although necrosis is not specific for fungal disease, hyphae may be sparse or obscured within necrotic debris. Histological assessment should document organism morphology, host response, and tissue or vascular invasion, while species-level identification generally requires microbiological or molecular correlation [8-10,14,15]. The detection of sparse fungal elements in eosinophilic mucin may depend on the staining method, tissue-processing approach, and fungal burden [11]. Previous reports have shown that fungal elements may occasionally be missed on routine stains, particularly in specimens with eosinophilic mucin or low fungal burden; therefore, the absence of PAS-positive fungi in H&E-unsuspected cases in this cohort should not be interpreted as exclusion of occult fungal infection. A modified PAS protocol applied to frozen sections has been reported to improve fungal visualization in suspected acute invasive fungal rhinosinusitis [17].

All PAS-positive fungal cases had already been suspected on H&E, and no unsuspected cases were identified. Because PAS slides were reviewed with knowledge of the corresponding H&E findings as part of the routine diagnostic workflow, and no independent reference standard (such as fungal culture, Gomori methenamine silver staining, molecular testing, or a composite reference standard) was available, this concordance should be interpreted as demonstrating the confirmatory utility of PAS rather than its independent diagnostic performance or evidence of 100% sensitivity, specificity, or diagnostic accuracy. GMS was not included in the present study because the primary objective was to evaluate the incremental utility of PAS within the routine diagnostic workflow rather than to compare fungal stains. GMS remains an important complementary stain, particularly for sparse, degenerated, or poorly visualized fungal elements; however, PAS may provide adequate confirmatory visualization in morphologically suspected cases. Allergic fungal rhinosinusitis and other non-invasive fungal conditions may demonstrate fungal hyphae without tissue necrosis, but no such cases were identified in the present cohort. Therefore, the absence of fungal-positive, non-necrotic cases should be interpreted as a finding specific to this cohort rather than evidence that non-invasive fungal disease does not occur. Future studies incorporating independent validation with GMS, fungal culture, molecular testing, or a composite reference standard are warranted to determine the diagnostic accuracy of PAS for fungal detection.

PAS-reactive mucin was identified in 119 cases, mainly in goblet cells and glandular secretions. Such changes are common in chronic rhinosinusitis and nasal polyps, but mucin demonstration did not alter diagnosis [5,6,18]. Routine PAS cannot reliably distinguish neutral mucin from glycogen or characterize acidic mucins. PAS-diastase, Alcian blue, mucicarmine, or other targeted stains may be preferable when characterization is important [7,19].

Only 34 neoplasms, including a few mucin-producing glandular tumors, were studied. Therefore, the limited neoplastic contribution should be interpreted cautiously. Sinonasal tumor diagnosis remains morphology-based, with immunohistochemical and molecular support where indicated [4]. A practical approach in routine practice may involve reviewing H&E first and considering PAS for cases with suspected fungi, necrotic or allergic mucinous debris, or a specific glandular component; however, this approach requires validation in studies incorporating blinded assessment.

Strengths and limitations

Strengths include consecutive case inclusion, a real-world tertiary-care specimen spectrum, and PAS staining in all eligible cases. Reanalysis of individual PAS components avoided treating the subjective overall intensity score as a diagnostic test and demonstrated a meaningful association between necrosis and fungal detection.

Limitations include the single-center design and the small number of neoplastic lesions. PAS slides were reviewed with knowledge of the corresponding H&E findings as part of the routine diagnostic workflow; therefore, the observed concordance between H&E suspicion and PAS demonstration of fungal elements should be interpreted as confirmatory rather than as an independent assessment of diagnostic performance, and observer bias cannot be excluded. Interobserver agreement was not assessed. Fungal findings were not systematically confirmed by fungal culture, Gomori methenamine silver (GMS) staining, immunohistochemistry, molecular methods, or another independent reference standard. Therefore, diagnostic accuracy estimates could not be calculated. PAS-diastase, Alcian blue, and mucicarmine were not evaluated. Subjective PAS intensity and eosinophil grading were excluded from inferential analysis. Lesion-wise and age-stratified subgroup analyses, as well as clinical follow-up, were beyond the scope of the present study. The study was designed to evaluate the overall contribution of PAS staining in consecutive sinonasal specimens rather than age-specific disease patterns, and the limited number of pediatric neoplastic lesions precluded meaningful age-stratified analyses.

## Conclusions

In this consecutive series of 162 sinonasal mass specimens, routine H&E morphology established the principal diagnosis in most cases. PAS did not substantially alter the initial histological categorization and did not reveal fungal organisms in cases without prior H&E suspicion. Its main value was the confirmatory demonstration of fungal structures in 19 cases with pre-existing morphological suspicion, particularly in specimens containing necrosis. PAS-reactive mucin was common but did not independently alter diagnosis. These findings support a morphology-guided approach to PAS utilization as a confirmatory ancillary stain in routine sinonasal histopathology practice; however, further studies with blinded assessment or independent validation are required to define its optimal role as a screening or confirmatory stain.
